# Biology of Tissue Inhibitor of Metalloproteinase 3 (TIMP3), and Its Therapeutic Implications in Cardiovascular Pathology

**DOI:** 10.3389/fphys.2020.00661

**Published:** 2020-06-16

**Authors:** Dong Fan, Zamaneh Kassiri

**Affiliations:** ^1^Department of Pathology, Zhuhai Campus of Zunyi Medical University, Zhuhai, China; ^2^Department of Physiology, University of Alberta, Edmonton, AB, Canada; ^3^Mazankowski Alberta Heart Institute, University of Alberta, Edmonton, AB, Canada

**Keywords:** tissue inhibitor of metalloproteinases, TIMP3, metalloproteinase, myocardial infarction, cardiovascular diseases, microRNA, extracellular matrix, gene therapy

## Abstract

Tissue inhibitor of metalloproteinase 3 (TIMP3) is unique among the four TIMPs due to its extracellular matrix (ECM)-binding property and broad range of inhibitory substrates that includes matrix metalloproteinases (MMPs), a disintegrin and metalloproteinases (ADAMs), and ADAM with thrombospondin motifs (ADAMTSs). In addition to its metalloproteinase-inhibitory function, TIMP3 can interact with proteins in the extracellular space resulting in its multifarious functions. TIMP3 mRNA has a long 3’ untranslated region (UTR) which is a target for numerous microRNAs. TIMP3 levels are reduced in various cardiovascular diseases, and studies have shown that TIMP3 replenishment ameliorates the disease, suggesting a therapeutic potential for TIMP3 in cardiovascular diseases. While significant efforts have been made in identifying the effector targets of TIMP3, the regulatory mechanism for the expression of this multi-functional TIMP has been less explored. Here, we provide an overview of TIMP3 gene structure, transcriptional and post-transcriptional regulators (transcription factors and microRNAs), protein structure and partners, its role in cardiovascular pathology and its application as therapy, while also drawing reference from TIMP3 function in other diseases.

## Introduction

The family of tissue inhibitor of metalloproteinases (TIMPs) is composed of 4 members, TIMP1 to TIMP4, which are the physiological inhibitors of matrix metalloproteinases (MMPs). The balance between MMPs and TIMPs plays a key role in the stability and normal function of the extracellular matrix (ECM). A disruption in this balance can cause fibrosis, excess accumulation of matrix proteins, or ECM degeneration, although the complexity of this process extends beyond the ability of the MMPs degrading the ECM proteins and TIMPs inhibiting this process (Takawale et al., 2015). TIMP3 is unique among the four TIMPs because it is the only one with a high affinity for the proteoglycans in the ECM, as well as its broadest range of substrates, including all MMPs, and a number of ADAMs (a disintegrin and metalloproteinases) and ADAMTSs (ADAM with thrombospondin motifs) (Staskus et al., 1991; Pavloff et al., 1992; Leco et al., 1994; Apte et al., 1995; Brew and Nagase, 2010; Moore et al., 2012; Jackson et al., 2017). Nevertheless, increasing number of reports show that TIMPs have more diverse functions beyond MMP inhibition. A variety of molecular partners for TIMP3 have also been reported that can mediate its MMP-independent functions (Qi et al., 2003; Klenotic et al., 2004; Nour et al., 2005; Kang et al., 2008; Scilabra et al., 2013; Huttlin et al., 2015, 2017; Kwasna et al., 2018).

Cardiovascular diseases continue to be a leading cause of morbidity and mortality (GBD 2017 Causes of Death Collaborators, 2018; GBD 2017 Disease and Injury Incidence and Prevalence Collaborators, 2018). Cardiac remodeling is a key characteristic of heart disease; it is the cumulative outcome of myocyte death (apoptosis and/or necrosis), myocyte hypertrophy, and fibrosis, and plays a pivotal role in progression to heart failure (Cohn et al., 2000; Fan et al., 2012; Moore et al., 2012). TIMP3 is involved in various process of cardiac remodeling and its level is reduced in ischemic and dilated cardiomyopathy (Li et al., 1998; Moore et al., 2012). Translational studies on the treatment of heart diseases such as myocardial infarction (MI) with TIMP3 replenishment suggest the therapeutic potential of TIMP3 in patients with heart failure (Spinale and Villarreal, 2014; Takawale et al., 2017). Recently, Su et al. (2019) summarized the roles and therapeutic potential of TIMP3 in cancer. Therefore, it will be valuable to compile the knowledge and advances in TIMP3 structure and function which will support the basis for further investigations in understanding its clinical application in cardiovascular diseases. In this review, we will focus on the roles of TIMP3 in cardiomyopathies, on its partners and applications.

## Partners of TIMP3 Gene and MRNA

### TIMP3 Gene

Tissue inhibitor of metalloproteinase 3 is widely expressed in various tissues at a relatively high level (Fagerberg et al., 2014; Uhlen et al., 2015). The genomic organization of TIMP3 contains 5 exons (Apte et al., 1995; Stohr et al., 1995). In human and mouse, TIMP3 gene is located in chromosome 10 and 22, respectively (Apte et al., 1994a, b). The TIMP3 gene (*Timp3*) is nested in an intron of the synapsin-3 gene (*Syn3*), but *Timp3* and *Syn3* are transcribed from different DNA strands, like most nested genes, and there is no evidence showing coordination of expression or biological function between these two genes (reviewed in Candiani et al., 2010; Brew, 2019). TIMP3 expression can be regulated at both transcriptional and post-transcriptional levels.

### Transcriptional Regulation of TIMP3 Expression

Transcriptional regulations are mainly mediated by transcription factors binding to *cis*-regulatory DNA elements in the promoter region of a gene (Inukai et al., 2017). The transcription initiation site is commonly referred to as the +1 position, and promoters can be located in its upstream (labeled as minus) and downstream (positive) sequence (Butler and Kadonaga, 2002). There are three conserved putative Spl consensus binding sites in both human and murine *Timp3* promoter, and a number of not highly conserved sites such as AP1, PEA3, and CTF/NF-1 binding sites (Stohr et al., 1995). *Timp3* expression can be upregulated by multiple cytokines and growth factors such as phorbol ester (PMA), epidermal growth factor (EGF), transforming growth factor-β1 (TGF-β1) (Leco et al., 1994), and bovine lactoferricin, a peptide derived from bovine lactoferrin (Yan et al., 2013). TGF-β1 and bovine lactoferricin induce *Timp3* expression through ERK1/2-activated Sp1 binding to its regulatory element in the *Timp3* promoter (Qureshi et al., 2005; Yan et al., 2013). TGF-β1 can also stimulate transcription of *Timp3* through activation of smad2/3/4 which bind to *Timp3* promoter regions −828 to −333 bp and −333 to +1 bp (Qureshi et al., 2008). Overexpression of Elf3a and Elf3b (two splice variants of ETS-related transcription factor Elf-3) enhances the activity of *Timp3* promoter by binding to the regions corresponding to −177 to −147 bp (human promoter) and −174 to −144 bp (murine promoter). However, Elf3a and Elf3b are normally expressed in rat lung, kidney, liver, and retina, but not in the heart (Jobling et al., 2002), therefore, their contribution to cardiac *Timp3* overexpression may be limited.

### MicroRNAs Bind to and Downregulate TIMP3

Among the four TIMPs, *Timp3* has the longest 3’ untranslated region (UTR) contained in the exon 5 (Leco et al., 1994; Apte et al., 1995), which is a target for multiple microRNAs. MicroRNAs (miRNA, miR) are a group of short, non-coding RNAs with 18−25 bases which can regulate gene expression at the transcriptional and post-transcriptional stages (Mohr and Mott, 2015; Dutka et al., 2019; Yao et al., 2019). MiRNAs have been shown widely to be involved in cardiovascular diseases (Barwari et al., 2016; De Rosa et al., 2017; Kir et al., 2018; Dutka et al., 2019). MiR-17-92 cluster is able to induce cardiomyocyte proliferation in postnatal and adult heart as well as in response to myocardial infarction (Chen et al., 2013), which is partly caused by binding of miR-17 to 3’ UTR of *Timp3* and suppressing *Timp3* expression (Shi et al., 2017; Liu et al., 2018). Through binding to, and inhibiting *Timp3 expression*, miR-21 enhances survival, migration and capillary formation ability of human umbilical vein endothelial cell lines (HUVECs) (Hu et al., 2016). miR-181b targets *Timp3* and reduces apoptosis of cardiomyocytes (Gao et al., 2017), but enhances gelatinases/MMP activity in human aortic valve endothelial cells which may contribute to calcific aortic valve disease (Heath et al., 2018). MiR-181b is increased in human atherosclerotic plaques and abdominal aortic aneurysms (AAA), and inhibition of miR-181b suppressed the development and progression of atherosclerosis and aneurysms through increasing expression of TIMP3, elastin, and collagen (Di Gregoli et al., 2017). Upregulation of miR-181b by TGF-β can also promote hepatocarcinogenesis via targeting TIMP3 (Wang et al., 2010). MiR-222 is required for exercise-induced cardiac growth and can protect the heart against adverse remodeling (Liu et al., 2015). MiR-222 has also been reported to target *Timp3*, thereby promoting the proliferation of pulmonary arterial smooth muscle cells (Xu et al., 2017). MiR-323a binds to and down-regulates *Timp3* which was associated with increased fibrosis following cardiac pressure overload (Zhang J. et al., 2018). *Timp3* expression can also be negatively regulated by miR-712 (human homolog, miR-205) (Son et al., 2013; Kim et al., 2014). And inhibition of miR-712 by Anti-miR-712 has been shown to suppress atherosclerosis, inflammation, and AAA induced by angiotensin II (Ang II) (Son et al., 2013; Kim et al., 2014), implying a protective role for TIMP3 in these processes. A summary of all microRNAs reported to date to target *Timp3* with specific binding sites, and the corresponding cell types are listed in Table 1.

**TABLE 1:** Functions of miRNAs targeting *Timp3* and their binding sites in 3′ UTR of *Timp3* mRNA.

miRNA	Cell/tissue	Binding site	Function	References
miR-17	Cardiomyocyte, H9C2, 293T	1351−1357	Hypertrophy↑, Proliferation↑, survival↑; TIMP3 ↓	Shi et al., 2017; Liu et al., 2018
	Prostate cancer cells, Bladder cancer cells	101−123, 524−545	Proliferation ↑, Survival ↑, migration ↑, invasion ↑, colony formation ↑, tumorigenesis ↑; TIMP3 ↓	Yang X. et al., 2013; Peng and Li, 2019
miR-21	HUVECs	1032−1039	Survival ↑, migration ↑, capillary formation ↑; TIMP3 ↓, MMP2 ↑, MMP9 ↑	Hu et al., 2016
	RCC cell, 293T,cervical cancer cell	1032−1039	Proliferation ↑, migration ↑, invasion ↑; TIMP3 ↓	Chen et al., 2017; Zhang Z. et al., 2018
	Podocytes, 293T	2322−2329	Apoptosis ↑, IL-1β↑, TNF-α↑, Bax ↑; TIMP3 ↓	Chen X. et al., 2018
miR-34b	MRC-5, 293T	1282−1288	Fibrosis ↑, col I ↑; TIMP3 ↓	Hu et al., 2019
miR-136	Neuronal cell	2582−2600	Apoptosis ↓; TIMP3 ↓	Jin et al., 2017
miR-142	DRG neuron cells, 293T	55−61	Viability ↑, apoptosis ↓; TIMP3 ↓	Wu et al., 2018
miR-181b	Cardiomyocytes, 293T	797−811	Apoptosis ↓; TIMP3 ↓	Gao et al., 2017
	HCC	3499−3505, 3573−3580	Growth ↑, clonogenic survival ↑, migration ↑, invasion ↑, tumorigenicity ↑; TIMP3 ↓, MMP2 ↑, MMP9 ↑	Wang et al., 2010
miR-191	Prostate cancer cell	3553−3573	Growth ↑, invasion ↑; TIMP3 ↓	Wang X. et al., 2018
miR-205 (miR-712)	iMAECs, VSMCs	3495−3500	AAA ↑, inflammation ↑, atherosclerosis ↑, VSMC migration ↑, EC permeability ↑; TIMP3 ↓, RECK ↓, MMP activity ↑, ADAM activity ↑, ADAMTS4 activity ↑	Son et al., 2013; Kim et al., 2014
miR-206	THP-1, CFb, 293T	1135−1146, 1683−1689	Inflammatory cytokines ↑; TIMP3 ↓, MMP2 ↑, MMP9 ↑	Limana et al., 2011; Fu et al., 2016
miR-221	PTC cell, 293T	2429−2450	Proliferation ↑, apoptosis ↓, migration ↑; TIMP3 ↓	Diao et al., 2017
miR-222	PASMCs, NSCLC, HCC, OS	2443−2449	Migration ↑, proliferation ↑, invasion ↑; TIMP3 ↓	Garofalo et al., 2009; Xu et al., 2017; Guo et al., 2018
miR-323a	CFb, 293T	3590−3596	Proliferation ↑, fibrosis ↑, Col I ↑, col III ↑, TGF-β↑; MMP2 ↑, MMP9 ↑, TIMP3 ↓	Zhang J. et al., 2018
miR-365	rMC-1, 293T	336−342	Gliosis ↑, oxidative stress ↑; TIMP3 ↓	Wang J. et al., 2018
miR-373	ESCC cell lines	112−118	Migration ↑, proliferation ↑, invasion ↑; TIMP3 ↓	Liu et al., 2016
miR-770	Podocytes	227−232	Apoptosis ↑, IL-1β↑, TNF-α↑; TIMP3 ↓	Wang and Li, 2020

*293T, human embryonic kidney 293T cell; AAA, abdominal aortic aneurysm; CFb, cardiac fibroblasts; col, collagen; DRG, dorsal root ganglion; ESCC, Esophageal squamous cell carcinoma; H9C2, rat heart myoblast H9C2 cells; HCC, hepatocellular carcinoma cells; HUVECs, human umbilical vein endothelial cells; iMAECs, immortalized mouse aortic endothelial cells; MRC5, human lung fibroblast line; NSCLC, non-small cell lung cancer cells; OS, osteosarcoma cell; PASMCs, pulmonary artery smooth muscle cells; PTC, papillary thyroid cancer; RCC cell, renal cell cancer cell; RECK, reversion-inducing cysteine-rich protein with kazal motifs; rMC-1, rat Müller cell line; THP-1, human THP-1 macrophages; VSMCs, vascular smooth muscle cells. ↑, increased or activated; ↓, decreased or inactivated.*

## Partners of TIMP3 Protein

### TIMP3 Protein Structure

**FIGURE 1:**
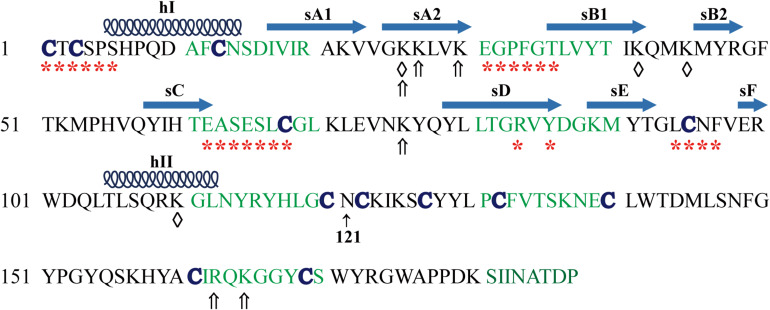
Mature human TIMP3 protein sequence and putative binding sites (domain) of TIMP3 with its interactors. hI-II, sA-F, location and extent of α-helices and β-strands as described (Wisniewska et al., 2008). Asn121 separates the N- and C-terminal domains of TIMP3. Partners of N-terminal TIMP3: ADAM17 (a disintegrin and metalloproteinase 17, *), ADAM12, MMPs (matrix metalloproteinases), ADAMTS (ADAM with thrombospondin motifs)-2, -4, -5, AT2R (angiotensin II type 2 receptor), GAGs (glycosaminoglycans, ⇑); LRP-1 [Low-density lipoprotein (LDL) receptor-related protein-1, ♢]. Partners of C-terminal TIMP3: MMP-2, -3, proMMP-2, -9, GAGs (⇑), fibulin-3, PC5A (a soluble isoform of proprotein convertase 5), VEGFR2 (vascular endothelial growth factor receptor 2).

Human and mouse TIMP3 protein precursor is composed of 211 amino acids with a secretion signal peptide (1−23 aa) followed by the mature TIMP3 protein sequence (Leco et al., 1994; Wilde et al., 1994). The mature TIMP3 protein is about 24 kDa (Figure 1), and has an N-linked glycosylation site (Asn184) near the carboxyl terminus, while the size of the glycosylated TIMP3 is about 27 kDa (Leco et al., 1994; Wilde et al., 1994; Apte et al., 1995; Langton et al., 1998). Two-thirds of the mature TIMP3 (∼120 aa) forms the N-terminal domain, and the remaining one-third constitutes the C-terminal domain. The secondary structure of TIMP3 is composed of 6 loops stabilized by 6 disulfide bonds formed by 12 conserved cysteine residues (Figure 1), which is a common feature in all four TIMPs (Wisniewska et al., 2008; Moore et al., 2012). The N- and C-terminal domains contain 3 loops each. The 3-dimentional structure of the N-terminal TIMP3 (N-TIMP3, Cys1-Asn121) exhibits a wedge-like shape and contains an oligosaccharide/oligonucleotide binding (OB) fold (consisting of 5 β–pleated strands, sA-sE), an N-terminal and a C-terminal α-helix (hI-hII, Figure 1; Wisniewska et al., 2008; Brew and Nagase, 2010; Brew, 2019).

### Interaction With Metalloproteinases

With the widest inhibitory spectrum against metalloproteinases, TIMP3 can suppress all MMPs, a number of ADAMs (−10, −12, −17, −28, and −33) and ADAMTS (−1, −2, −4, and −5) (Brew and Nagase, 2010; Moore et al., 2012; Jackson et al., 2017; Brew, 2019). ADAM17 is inhibited by TIMP3 when present as a dimer; and the N-terminal domain of TIMP4 can interact with and inhibit ADAM17 *in vitro*, suggesting that the C-terminal domain of TIMP4 may disrupt this interaction *in vivo* (Amour et al., 1998; Lee et al., 2005; Brew and Nagase, 2010). Full-length and N-terminal (1−115 aa) TIMP3 demonstrate equal binding and inhibitory efficacy toward ADAM17 (Lee et al., 2001), suggesting that there is little influence of C-terminal domain of TIMP3 on its binding to ADAM17. TIMP3 interacts with the catalytic domain of ADAM17 (Arg215-Val477) via five segments as follows (Figure 1; Wisniewska et al., 2008): (1) major contacts by binding of Cys1-Ser6 of TIMP3 into the non-primed side of the active-site cleft of ADAM17, and the Cys1 directly above the catalytic zinc ion of ADAM17; (2) sA-sB hairpin loop of N-TIMP3 (Glu31-Gly32-Pro33-Phe34-Gly35- Thr36) extending into a hydrophobic groove formed by the side chains of ADAM17 residues Tyr352, Val353, Tyr369 and Leu380, which maintains the stability of TIMP3^∗^ADAM17 complex; (3) the sC-connector loop (Glu62-Ala63-Ser64-Glu65- Ser66-Leu67-Cys68) of TIMP3 with ADAM17 residues Lys315, Leu350-Tyr352 and His415; (4) Arg84 and Tyr86 of the sD-sE loop of TIMP3 with Asp313, Met316/Pro305 of ADAM17; (5) Multiple contacts of the sE-sF loop of N-TIMP3 with the catalytic domain of ADAM17 (Pro437 and His415). However, the extracellular form of ADAM17 (215−651 aa) is more resilient to N-TIMP3 than the catalytic domain of ADAM17 (215−477 aa), indicating that C-terminal domains of ADAM17, which contain disintegrin, EGF-like and Crambin-like domains, significantly weaken the inhibitory effect of N-TIMP3 (Lee et al., 2002).

Xu et al. (2012) reported that ADAM17 dimer has a greater capacity to interact with TIMP3, whereas activation of p38 or ERK1/2 reduces the dimerization of ADAM17 and its association with TIMP3, therefore increasing activation of ADAM17. Since a 1:1 stoichiometry has been reported for the TIMP3^∗^ADAM17 complex (Wisniewska et al., 2008), two TIMP3 protein would interact with the ADAM17 dimer. Because ADAM17 is a very important proteinase that can cleave numerous membrane-bound cytokines and growth factors (or their receptors) including the full length TNF-α (reviewed in Zhang et al., 2016; Zunke and Rose-John, 2017), TIMP3 has a variety of functions besides the anti-inflammatory effect through inhibition of ADAM17. The N-terminal domain of TIMP3 (N-TIMP3) also binds to the catalytic domain of ADAM12 and deletion of the disintegrin and cysteine-rich domains in ADAM12 enhanced the inhibitory effect of TIMP3 against this ADAM (Jacobsen et al., 2008; Kveiborg et al., 2010). However, N-TIMP3 is insufficient for binding to and inhibiting ADAM10, whereas full length TIMP3 is an efficient inhibitor of ADAM10 (Rapti et al., 2008).

The interaction and inhibitory effect of TIMP3 on MMPs are similar to the N-TIMP3^∗^ADAM17 and MMP^∗^TIMP1 complex, involving the ridge formed by N-TIMP3 residues Cys1-Thr2-Cys3-Ser4-Pro5, sC-sD loop (Ser64-Glu-Ser-Leu- Cys68) and the Cys1-Cys68 disulfide bond, which inserts into the MMP active site and the Cys1 lies above the catalytic zinc ion of MMPs (Wisniewska et al., 2008; Brew and Nagase, 2010). Mutation of Cys1 to Ser in TIMP3 results in loss of its MMP-inhibitory activity (Bond et al., 2000).

In the C-terminal domain of TIMP3, a mutation of Ser181 to Cys seen in Sorby’s fundus dystrophy (SFD), or N-glycosylation (Asn184) have no effect on TIMP3 inhibitory function against MMPs (Langton et al., 1998). In addition, the C-terminal domain of TIMP3 can interact with MMP2 and MMP3 which assists the MMP-inhibitory effects of TIMP3 (Troeberg et al., 2009). The C-terminal domain of TIMP3 can also bind to the hemopexin domains of proMMP2 and proMMP9 (Butler et al., 1999). TIMP3 has been reported to assist the activation of proMMP2 by membrane-type-3 MMP (MT3-MMP) through forming a trimolecular complex with proMMP2 and MT3-MMP (Zhao et al., 2004). The activation of proMMP2 by MT3-MMP is enhanced by TIMP3 in a dose-dependent fashion occurring at 100 nM, but not at 1−30 nM which in fact inhibited formation of the intermediate form of MMP2 (Zhao et al., 2004). TIMP3 has been reported not to affect the activation of proMMP2 by MT1-MMP (Zhao et al., 2004), while TIMP3-deficiency promotes activation of proMMP2, which can be inhibited by N-TIMP3 (10−50 nM) but not matrix-associated TIMP3 (English et al., 2006).

The N-terminal domain of TIMP3 can also interact with and inhibit ADAMTS-2, -4 and -5 (Kashiwagi et al., 2001; Gendron et al., 2003; Wang et al., 2006). The TIMP3 inhibitory site interacting with ADAMTS4 is composed of N-terminal tail (Cys1-Thr2-Cys3-Ser4-Pro5), an inhibitory loop (sC-connector loop) formed by Glu62-Ala63-Ser64-Glu65-Ser66-Leu67-Cys68, and the Cys1-Cys68 disulfide bond (Zhang W. et al., 2018). The inhibitory loop directly inserts into the catalytic center of ADAMTS4 which plays the primary role, whereas the N-terminal tail assists the interaction and forms a coordinate bond between the carbonyl group of Cys1 and the zinc ion chelator (Zhang W. et al., 2018). The C-terminal domains of ADAMTS4 and ADAMTS5 can enhance their association with N-TIMP3 (Troeberg et al., 2009).

### Interaction With LRP-1 and TIMP3 Turnover (Degradation)

Low-density lipoprotein (LDL) receptor-related protein-1 (LRP-1) belongs to the LDL receptor family which mediate endocytosis and degradation of multiple ligands (Strickland et al., 2002). It has been reported that LRP-1 could directly bind to TIMP-1, -2, and -3, and mediate their endocytosis and degradation (Emonard et al., 2004; Troeberg et al., 2008; Scilabra et al., 2013; Thevenard et al., 2014). Heparin, pentosan polysulfate, or receptor-associated protein (RAP), an antagonist of ligands binding to LRP family members, result in accumulation of TIMP3 in the culture medium (Troeberg et al., 2008). Scilabra et al. (2013) reported that TIMP3 binds to the extracellular domain of LRP-1, while a soluble form of LRP-1 (sLRP-1) shed from the cell membrane can also bind to TIMP3 leading to its accumulation in the medium since it cannot be endocytosed. This group further reported that TIMP3 binds to the N-terminal domain of the ligand-binding cluster II of LRP-1 (sLRP2N), which is not influenced by interaction of LRP-1 with MMP-13 or ADAMTS-5 (Yamamoto et al., 2016; Scilabra et al., 2017). LRP-1 can mediate the endocytosis of TIMP3, MMP-13 and ADAMTS-5 at the same time. The sLRP2N without the EGF-like repeat can be used as a TIMP3 trap to sequester TIMP3 in the culture medium (*in vitro*) or in the extracellular space (*in vivo*) which supresses metalloproteinase-mediated shedding of various cytokines and receptors (Scilabra et al., 2017). Blocking LRP-1 with RAP or an LRP-1 blocking antibody could also raise the level of TIMP-3 on the surface of endotoxin-activated macrophages and suppress the release of TNF by ADAM17 (Schubert et al., 2019). Mutation of two lysine pairs of TIMP3 (K26A/K45A or K42A/K110A, Figure 1) disrupts its interaction with LRP-1 and prevents its endocytosis (Doherty et al., 2016). This can be employed as an approach to accumulate TIMP3 in the extracellular space and thereby inhibit excess metalloproteinase activity.

### Interaction With ECM

Wildtype TIMP3 and a TIMP2-TIMP3 chimera created by fusing the N-terminal domain of TIMP2 to the C-terminal domain of TIMP3 are predominantly found in the ECM, whereas N-TIMP3 without the C-terminal domain was almost undetectable in the COS-7 cell ECM (Leco et al., 1994; Langton et al., 1998). Later, it was demonstrated that both N- (19-32 aa and 41-52 aa) and C-terminal domains participate in the binding of TIMP3 to ECM proteins (Yu et al., 2000; Lee et al., 2007). The residues of TIMP3 involved in this binding are mainly basic amino acids: Lys26, Lys27, Lys30, and Lys76 of the N-terminal domain, Arg163 and Lys165 of the C-terminal domain (Figure 1). Replacement of these residues with Glu and Gln deprives TIMP3 of its ECM-binding ability and renders it soluble (Lee et al., 2007). Both glycosylated and non-glycosylated TIMP3 can bind to ECM (Apte et al., 1995; Langton et al., 1998). The specific ECM proteins interacting with TIMP3 are mainly several glycosaminoglycans (GAGs): heparin, heparan sulfate (HS), chondroitin sulfates, and sulfated compounds such as suramin and pentosan, but not dermatan sulfate, aggrecan or hyaluronic acid (Yu et al., 2000; Troeberg et al., 2014). The C-terminal domain of TIMP3 can also bind to another matrix protein fibulin-3, known as EGF-containing fibulin-like extracellular matrix protein 1 (Klenotic et al., 2004).

Preincubation of TIMP3 with heparin, HS or chondroitin sulfate E (CSE) inhibits its binding to the ectodomain of LRP-1, and subsequently its endocytosis by LRP-1 (Troeberg et al., 2008, 2014; Scilabra et al., 2013). Treating cells in culture with HS or CSE also leads to the accumulation of TIMP3 in culture media. HS and CSE can enhance the inhibitory effects of TIMP3 on ADAMTS-5 by increasing TIMP3 affinity for ADAMTS-5 and reducing the dissociation rate (Troeberg et al., 2014). Therefore, ECM is not only a depot for TIMP3, but also protects TIMP3 against endocytosis and degradation, enhances the binding of TIMP3 to metalloproteinases and facilitates its inhibitory function.

### Interaction With VEGFR2

Tissue inhibitor of metalloproteinase 3 has been reported to suppress vascular endothelial growth factor (VEGF)-mediated angiogenesis through directly binding to VEGF receptor 2 (VEGFR2) and disrupting the interaction of VEGF with VEGFR2 and the downstream signaling (Qi et al., 2003; Janssen et al., 2008). TIMP3 can also bind to soluble VEGFR2 (sVEGFR2), suggesting that TIMP3 binds to the extracellular domain of VEGFR2 (Qi et al., 2003). Two synthetic peptides derived from the loop 6 (aa 137−166) and tail region (aa 167−188) or C-terminal domain (aa 122−188) of TIMP3 mimic the interaction of TIMP3 and VEGFR2 (Janssen et al., 2008; Qi et al., 2013). Therefore, the C-terminal domain of TIMP3 has been proposed to be responsible for its interaction with VEGFR2. An SFD mutation S156C did not alter the interaction between TIMP3 and VEGFR2, or its anti-angiogenic effect (Fogarasi et al., 2008). However, TIMP3 (S156C) expression in porcine aortic endothelial cells enhanced VEGF binding to VEGFR2 and VEGF-induced angiogenesis through up-regulation of VEGFR2 (Qi et al., 2009), which might lead to choroidal neovascularization in SFD. The discrepancy between these two studies is probably due to the up-regulation of VEGFR2 in porcine aortic endothelial cells expressed with TIMP3 (S156C) and the levels of TIMP3 (S156C) involved. The up-regulation of VEGFR2 was also seen in human eyes with SFD (Qi et al., 2009). Meanwhile, this TIMP3-VEGR2 interaction has been reported to occur only at high concentrations of TIMP3; such that at physiological concentrations, TIMP3 does not interact with VEGFR2, and in fact promotes angiogenesis, but at high concentrations, TIMP3 can suppress angiogenesis by binding to VEGR2 and blocking the downstream pathway (Takawale et al., 2017). TIMP3 inhibits VEGF binding to VEGFR2 with a half-maximal inhibitory concentration (IC_50_) of 3.3−4.5 μg/ml, whereas cold VEGF competes with labeled VEGF for binding to VEGFR2 with an IC_50_ of 8.5−15 ng/ml (Qi et al., 2003). Therefore, the affinity of VEGFR2 for TIMP3 is much lower than its affinity for VEGF. However, the reason of pro-angiogenesis by low concentration of TIMP3 remains unknown.

### Interaction With AT2R

Both Ang II type 2 (AT2R) and type 1 (AT1R) receptors contain seven-transmembrane domains and belong to the G-protein-coupled receptor (GPCR) family (Verdonk et al., 2012). TIMP3 can interact with AT2R but not AT1R (Kang et al., 2008). The interaction is mediated by the N-terminal domain of TIMP3 and the C-terminal sequence (aa 235−363) of AT2R (Kang et al., 2008). Overexpression of TIMP3 or AT2R have no obvious effect on normal cells (HEK 293 cells or HUVECs), but each of them can induce the apoptosis of SKOV-3 ovarian cancer cells through activation of caspase-3 and -9, and collectively they have additive effects (Kang et al., 2008). Overexpression of TIMP3 or AT2R inhibits VEGF-induced angiogenesis, and their combined overexpression has an additive effect; however, silencing AT2R with siRNA does not block the effect of TIMP3, and vice versa (Kang et al., 2008), which suggest that the anti-angiogenic effect of TIMP3 may not be mediated by AT2R. As discussed above, TIMP3 may suppress VEGF-induced angiogenesis through binding to and inhibiting VEGFR2 signaling. Although the specific binding site for TIMP3 on AT2R requires further investigation, AT2R binds to the N-terminal domain of TIMP3, while VEGFR2 binds to the C-terminal domain of TIMP3, therefore, TIMP3 may be able to bind to VEGFR2 and AT2R at the same time. The functional outcome of TIMP3 binding to AT2R remains largely unknown. Due to vital roles of AT2R and AT1R in the cardiovascular system, it is worth exploring the implications of this interaction in cardiovascular diseases.

### Interaction With Other Proteins

The proprotein convertase 5 (PC5) is a serine proteinase that cleaves numerous proproteins at paired basic amino acids within the secretory pathway (Seidah and Chretien, 1999). The C-terminal cysteine-rich domain (CRD) of PC5A, a soluble isoform of PC5, can bind to the C-terminal domain of all four TIMPs (Nour et al., 2005). TIMP3 can also interact with a de-ubiquitinating enzyme ZUFSP which plays an important role in genomic stability (Kwasna et al., 2018). By using high-throughput affinity purification-mass spectrometry methodology, Huttlin et al. (2015, 2017) found other TIMP3 partners such as C-reactive protein (CRP), TIMP2, ADP-dependent glucokinase (ADPGK), Asialoglycoprotein receptor 2 (ASGR2), C-C motif chemokine 3-like 1 (CCL3L1), collectin-11, Dickkopf-related protein 3 (DKK3), neutrophil defensin 1 (DEFA1), gamma-interferon-inducible lysosomal thiol reductase (IFI30), Insulin-like growth factor-binding protein 1 (IGFBP1), von Willebrand factor C and EGF domain-containing protein (VWCE). However, the specific binding sites and functional consequences demand further investigation.

In summary, in addition to inhibiting metalloproteinases, TIMP3 has several important roles in regulation of multiple molecules in the extracellular space and on the cell membrane, and the corresponding downstream intracellular signaling pathways, which could explain the diverse functions of this molecule in various pathologies. Here, we will summarize the reports on the role of TIMP3 in cardiovascular diseases and its potential as a therapeutic factor.

## TIMP3 in Cardiovascular Pathologies

### TIMP3 in Myocardial Disease

Tissue inhibitor of metalloproteinase three levels have been reported to be altered in patients with different cardiomyopathies. In patients with aortic stenosis, myocardial TIMP3 mRNA and protein, TIMP2 mRNA and TIMP4 protein are increased, while TIMP1 mRNA and protein decreased (Fielitz et al., 2004). In patients with atrial fibrillation, the myocardial TIMP3 protein level is increased (Mukherjee et al., 2006) or unchanged in another study (Polyakova et al., 2008), while the myocardial TIMP1 protein level is increased in both studies. In myocarditis, only the myocardial levels of TIMP3 (among the four TIMPs) are significantly increased (Polyakova et al., 2011). In the failing heart of patients with ischemic cardiomyopathy (ICM) or dilated cardiomyopathy (DCM), TIMP1 and TIMP3 levels were reduced with no change in TIMP2 and TIMP4, except TIMP4 protein decreased in ICM myocardium (Li et al., 1998); however, myocardial levels of all four TIMPs were increased in another study (Polyakova et al., 2011). Following mechanical unloading of the ICM or DCM failing hearts with LV assist device, myocardial levels of TIMP1 and TIMP3 were increased while no change in TIMP2 and TIMP4 (Li et al., 2001). Therefore, there is some discrepancy in how TIMP levels are altered in patients with myocardial disease, which could be related to the stage of disease, the region of the heart where the specimen was collected, the ethnic background of the patients, to name a few.

**FIGURE 2:**
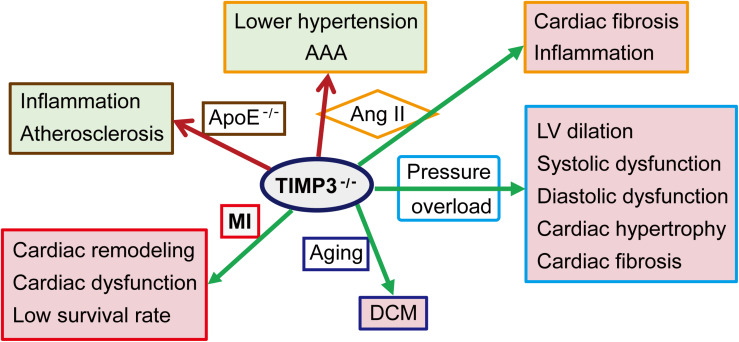
Role of TIMP3 in cardiovascular pathologies. TIMP3 deficiency (TIMP3^– /–^) exacerbates the structure change and dysfunction of the heart and/or the artery in a variety of animal models. AAA, abdominal aortic aneurysm; Ang II, Angiotensin II; ApoE^– /–^, apolipoprotein E-deficient mice; DCM, dilated cardiomyopathy; LV, left ventricular; MI, myocardial infarction.

The causal role of TIMP3 in progression of myocardial disease has been extensively explored in animal models (Figure 2). *Timp3* deficiency results in DCM in aged mice due to enhanced MMP9 activity and the activation of the TNF-α cytokine system (Fedak et al., 2004). Compared with wild-type (WT) mice, cardiac pressure overload in *Timp3*-knockout (*Timp3*^–/–^) mice leads to more severe LV dilation, systolic and diastolic dysfunction, cardiac hypertrophy and fibrosis, with early heart failure (Kassiri et al., 2005, 2009). This phenotype is caused by excess ADAM17 and TNF-α, as well as activation of MMP2 and MT1-MMP. Suppression of both TNF-α and MMPs was found to be necessary for the complete prevention of DCM in *Timp3*^–/–^ mice following pressure overload (Kassiri et al., 2005). Simultaneous activation of TNF-α and TGF-β1, and reciprocally stimulatory effects between these two factors, also played important roles in TIMP3-deficiency-induced adverse cardiac remodeling in the pressure-overload mice model (Kassiri et al., 2009). Other studies (Inagaki et al., 1995; Bitzer et al., 2000) had shown that TNF-α could inhibit TGF-β1 signaling, which might restrain signaling thresholds of TGF-β1, depending on physiological requirements. In addition to pressure overload, Ang II infusion induces severe fibrosis in *Timp3*^–/–^ mice, although in the absence of hypertrophy, mainly due to post-translational stabilization and deposition of collagen by matricellular proteins (Fan et al., 2014). Following myocardial infarction (MI), TIMP3 levels are decreased in the infarct and peri-infarct myocardium (Kandalam et al., 2010; Takawale et al., 2017). *Timp3*-deficiency exacerbates cardiac remodeling, dysfunction, and survival following MI (Figure 2), partly through increased MMP activity, TNF-α signaling, and apoptosis in the non-infarcted myocardium, while coronary density was not reduced in the myocardium (Tian et al., 2007; Kandalam et al., 2010). Further, a marked but transient rise in MMP activity in the early post-MI phase was found to be responsible for the adverse remodeling and increased rate of LV rupture in *Timp3*^–/–^ mice, since early treatment with a broad-spectrum MMP inhibitor ameliorated the adverse LV remodeling and rupture (Kandalam et al., 2010). On the other hand, targeted overexpression of TIMP3 or delivery of recombinant TIMP3 protein into the myocardium also improved the infarcted myocardium and heart function through suppression of the MMP activity and inflammation, and promoting angiogenesis (Eckhouse et al., 2014; Purcell et al., 2014, 2018; Barlow et al., 2017; Takawale et al., 2017; Chintalgattu et al., 2018; Thorn et al., 2019).

Since *in vitro* experiments lack the multicellular interactions that exist *in vivo*, the results from *in vitro* studies may differ from those *in vivo*. An *in vitro* study showed that silencing TIMP3 reduces apoptosis of cardiomyocytes isolated from 5 to 6 week-old female rats (Gao et al., 2017). However, deficiency or inhibition of TIMP3 enhances neonatal mouse cardiomyocyte proliferation *in vitro* and *in vivo*, which can be inhibited by treatment with recombinant TIMP3, through a mechanism involving inhibition of MMP activity and EGFR/JNK/SP-1 signaling pathway (Hammoud et al., 2009; Shi et al., 2017). The different findings from these studies could be caused by the difference in the age of the animals used for cardiomyocyte isolation. TIMP3 deficiency also promotes Ang II- or phenylephrine-induced fibrotic response in the co-culture of neonatal cardiac myocytes and fibroblasts, which may result from elevated levels and activity of metalloproteinases, and the subsequent proteolytic processing and activation of TGF-β1 and TNF-α (Kassiri et al., 2009).

### TIMP3 in Cardiac Inflammation

Tissue inhibitor of metalloproteinase 3 has been proposed to function as a physiological anti-inflammatory molecule primarily through its inhibitory ability against ADAM17, whereby it reduces the ADAM17-mediated release of soluble TNF-α, an important inflammatory cytokine (Khokha et al., 2013). Reduced MMP inhibition also contributes to migration of inflammatory cells in TIMP3 deficient mice (Moore et al., 2012; Khokha et al., 2013). TIMP3 deficiency enhances TNF-α levels and infiltration of neutrophils in the infarcted myocardium (Tian et al., 2007; Kandalam et al., 2010). Myocardial neutrophils and macrophages are also increased in TIMP3-deficient mice following Ang II infusion (Figure 2), which could be the cell source for the elevated matricellular proteins in these mice (Fan et al., 2014).

### TIMP3 in Aortic Aneurysm

Tissue inhibitor of metalloproteinase 3 levels in the aorta are decreased in patients with thoracic aortic aneurysm (Blunder et al., 2012; Lee et al., 2014), and in patients with acute type A aortic dissection (Kimura et al., 2017). *Timp3*-deficiency suppressed the hypertensive response to Ang II (Basu et al., 2013), however induced abdominal aortic aneurysm (AAA) after 4 weeks of Ang II infusion (Figure 2), due to degradation of the elastin fibers and adverse remodeling of the aortic wall (Basu et al., 2012). Ang II-mediated AAA was characterized by enlarged lumen, reduced aortic wall thickness due to loss of elastic lamellae, and inflammation (Basu et al., 2012). On the other hand, Ang II can suppress the expression of TIMP3 and reversion-inducing cysteine-rich protein with Kazal motifs (RECK) by upregulating miR-712 (human/murine homolog miR-205) (Kim et al., 2014). Silencing miR-712 and miR-205 suppressed MMP activity and inflammation, preventing Ang II-induced AAA. Therefore, TIMP3 and targeting miR-712 (miR-205) could be potential therapeutic strategies for AAA.

### TIMP3 in Angiogenesis

Recombinant TIMP3 protein suppressed angiogenesis by binding to and inhibiting VEGFR2 with an IC50 of 2.9 μg/ml (Qi et al., 2003; Janssen et al., 2008). However, this function of TIMP3 was later found to be dose-dependent such that at lower (close to physiological) concentrations (0.01 and 0.1 μg/ml), rTIMP3 promoted endothelial sprouting and angiogenesis, but at higher concentrations (>1 μg/ml), rTIMP3 blocked angiogenesis by binding to VEGFR2 and blocking the downstream signaling pathways (Takawale et al., 2017). Overexpression of TIMP3 and AT2R can additively inhibit VEGF-induced angiogenesis (Kang et al., 2008), implying the importance of both VEGFR2 and AT2R in angiogenesis.

### TIMP3 in Atherosclerosis

Atherosclerosis is described as a chronic inflammatory condition of the arterial system, and the thrombotic complications can cause MI, ischemic stroke, and peripheral arterial diseases, which can be potentially prevented by anti-inflammatory therapy (Libby et al., 2002; Geovanini and Libby, 2018). In patients with atherosclerosis, elevated TIMP3 levels in the plasma have been reported (Zheng et al., 2018), but TIMP3 is reduced in carotid atherosclerotic plaques from patients with type 2 diabetes (Cardellini et al., 2009). Deficiency of TIMP3 enhances inflammation and aggravates atherosclerosis in ApoE-knockout mice (Figure 2; Stohr et al., 2014), while overexpression of TIMP3 in macrophages decreases atherosclerosis (Casagrande et al., 2012). TIMP3 and RECK have also been reported to mediate the inhibitory effects of interleukin-32α (IL-32α) on endothelial inflammation, smooth muscle cell activation, and atherosclerosis development (Son et al., 2017). IL-32α upregulates TIMP3 and RECK by inhibiting miR-205 biogenesis. These data suggest that TIMP3 could exert anti-atherosclerotic effects.

## Application of TIMP3 in Cardiovascular Diseases

Due to the important role of TIMP3 in cardiac structure and function, and the severe adverse outcomes of *Timp3*-deficiency in heart disease (Fedak et al., 2004; Kassiri et al., 2005; Kandalam et al., 2010; Basu et al., 2012, 2013; Moore et al., 2012; Fan et al., 2014), treatment with TIMP3 could be a potential therapeutic method in these diseases. In fact, there have been several studies on the application of TIMP3 in heart disease (Table 2). Various delivery routes have been employed to replenish the TIMP3 levels in the diseased heart, each with advantages and disadvantages as described below.

**TABLE 2:** Application of TIMP3 in cardiovascular diseases.

Methods	Delivery route	Dose	Model	Function	References
Overexpression of TIMP3 with adenovirus	Intramyocardial injection (peri-infarct area)	5.5 × 10^7^ pfu/heart	MI in mice	Inhibited MMP activity, promoted angiogenesis, reduced MI expansion and ECM disruption, improved cardiac function at 1 week post-MI	Takawale et al., 2017
Cells -based TIMP3 (cells transfected with TIMP3 plasmid) delivery	Intramyocardial injection (infarct and peri-infarct area)	3 × 10^6^ Rat aortic VSMCs in 0.3 mL DMEM	MI in rats	Reduced MMP-2, MMP-9 and TNF-α levels, scar expansion and ventricular dilatation; improved LV systolic function at 4 weeks post-MI	Jia et al., 2014
	Intramyocardial injection (infarct area)	3 × 10^5^ Mouse BMSCs in 15 μL IMDM	MI in mice	Reduced early MMP activities, TNF-α levels, border zone apoptosis, scar expansion, ventricular dilatation; preserved remote myocardial collagen; improved LV systolic function at 4 weeks post-MI	Angoulvant et al., 2009
	Intramyocardial injection (infarct and peri-infarct area)	2 × 10^6^ Human MSCs in 100 μL IMDM	MI in rats	Increased angiogenesis, prevented adverse matrix remodeling, reduced infarct size, improved cardiac function at 4 weeks post-MI	Yao et al., 2012
Injectable hydrogels for TIMP3 delivery	Intramyocardial injection (infarct area)	20 μg rTIMP-3 in 100 μl hydrogels/injection/9 injection sites	MI in pigs	Reduced MMP activity, proinflammatory factors, LV dilation, and MI expansion; improved LV function during 14 days and up to 28 days post-MI	Eckhouse et al., 2014; Purcell et al., 2014, 2018
Intracoronary delivery of TIMP3	Intracoronary infusion (I/R area)	30 mg (or 1 mg/kg pig) rTIMP3	I/R in pigs	Reduced MMP activity, MI expansion and LV dilation; improved LV function at 28 days post-I/R	Barlow et al., 2017; Thorn et al., 2019
Overexpression of TIMP3 in vein graft with adenovirus	Vein graft infected with RadTIMP3	2.5 × 10^9^ pfu/mL RadTIMP3	A-V graft in pigs	Inhibited MMP activity and induced VSMC apoptosis, reduced neointima formation and restenosis at 28 days and 3 months after the infection and implantation	George et al., 2000, 2011
Utility of modified TIMP3 proteins	Intramyocardial injection (infarct area)	2 mg/rat TIMP3v2 or TIMP3v82	MI in rats	Suppressed MMP2/9 and ADAM17 activities, reduced LVEDV and LVESV, increased EF at 3 days (both) and 7 days (TIMP3v82) post-MI	Chintalgattu et al., 2018

*A-V graft, arteriovenous interposition grafting; BMSCs, Bone marrow stromal cells; DMEM, Dulbecco’s modification of Eagle’s medium; EF, ejection fraction; I/R, ischemia/reperfusion; IMEM, Iscove’s modified Dulbecco’s medium; MI, myocardial infarction; LV, left ventricular; LVEDV, LV end diastolic volume; LVESV, LV end systolic volume; MSCs, Mesenchymal stem cells; Pfu, plaque-forming units; RadTIMP3, recombinant adenovirus overexpressing TIMP3; rTIMP3, recombinant TIMP3 protein; TIMP3v2, TIMP3v82, two mutated TIMP3 proteins with extra glycosylation sites, TIMP3v2 (K22S/F34N) and TIMP3v82 (H55N/Q57T/K71N/E73T/D87N/K89T/R115T); TNF-α, tumor necrosis factor alpha; VSMCs, vascular smooth muscle cells.*

### Targeted Overexpression of TIMP3 With Viral Vectors

Viral delivery of a gene of interest has been achieved by adenovirus, adeno-associated virus (AAV), and lentivirus vectors as they allow for a broad host range. In a mouse model of MI, human TIMP3-expressing adenovirus (Ad-hTIMP3) was introduced into the peri-infarct myocardium by direct injections using a 36-gauge needle, increased TIMP3 mRNA and protein could be detected in the infarct and peri-infarcted myocardium (Takawale et al., 2017). This TIMP3 replenishment ameliorated cardiac dysfunction and the adverse remodeling post-MI, probably through inhibition of MMP activity and promotion of angiogenesis at 1 week post-MI, which is unexpected given the reports on anti-angiogenic functions of TIMP3 (Qi et al., 2003, 2013). Interestingly, an increased dose of Ad-hTIMP3 (5.5 × 10^8^ pfu/heart) expression exerted negative effects on myocardial remodeling and function post-MI (Takawale et al., 2017). This observation demonstrates the important notion that attempts toward overexpressing TIMP3, or any other TIMP, should be only to achieve their physiological levels and it should not be in excess.

Compared with the adenovirus-mediated overexpression, AAV vectors mediate long term (more than 1 year in non-proliferating cells), cardiac-specific gene expression, with much less to no pathogenic response and cytotoxicity in human hosts (Chu et al., 2003; Belke et al., 2006; Bera and Sen, 2017; Bass-Stringer et al., 2018; Ishikawa et al., 2018; Ambrosi et al., 2019). Systemic delivery of the AAV serotype 9 (AAV9) resulted in transduction of myocytes throughout the myocardium, but caused cardiac fibrosis and dysfunction to some degree (Merentie et al., 2016), suggesting that targeted myocardial injection is perhaps a safer mode of delivery, although given the invasive nature of this approach which can pose limitations.

### Cell-Based TIMP3 Delivery

Cell-based gene therapy for MI is a valid approach since cardiomyocytes and vascular cells are lost in the process of infarct formation (Chen Z. et al., 2018). Intramyocardial injections of rat aortic VSMCs, mouse bone marrow stromal cells, or human mesenchymal stem cells transfected with a TIMP3 plasmid have been reported to be more effective in improving cardiac structure and function post-MI than the cells without transfection (Angoulvant et al., 2009; Yao et al., 2012; Jia et al., 2014). Therefore, cell-based TIMP3 delivery could be an efficient approach for MI since TIMP3 can inhibit metalloproteinases and activation of TNF-α, as well as promote angiogenesis, and further improve the structure and function of the heart post-MI. However, transplantation with exogenous cells may provoke immune reaction in the host, whereas employing autologous cells transfected with the gene of interest *in vitro* prior to transplantation has been proposed to provide a better option, but it requires 3 or more days for the newly introduced genes to be expressed before the transplant procedure can be completed (Jia et al., 2014; Melly et al., 2018). Thus it could be used for long term treatment effects to prevent progression of infarct expansion and cardiac dysfunction post-MI. However, the long term effects of this approach needs to be determined.

### Injectable Hydrogels for TIMP3 Delivery

Local application of recombinant TIMP3 protein (rTIMP3) with hydrogels can also reduce myocardial remodeling and improve heart function post-MI (Eckhouse et al., 2014; Purcell et al., 2014, 2018). Hydrogels based on hyaluronic acid (HA) containing full-length human rTIMP3 were injected into the myocardium in an MI model in pigs (Eckhouse et al., 2014). When not bound to HA (Troeberg et al., 2014), TIMP3 can be released from the hydrogel at a nearly uniform rate for up to 14 days. After 7 days post-injection, human TIMP3 protein could be detected in the targeted myocardium, but not in the non-target myocardium or other organs (Eckhouse et al., 2014). An injectable and MMP degradable hydrogel has also been developed to contain rTIMP3 for “on-demand” MMP inhibition (Purcell et al., 2014). Dextran sulphated (DS), a heparin mimetic sulfated GAGs which can bind to TIMP3, crosslinks with HA hydrogels via MMP-cleavable peptide, so that rTIMP3 is encapsulated in the hydrogels until MMPs present and degrade the hydrogel (Purcell et al., 2014).

With their dual forms, liquid at room temperature (20−25°C) and gelatin-like at body temperature (37°C), the injectable hydrogels are attractive media for drug release and tissue engineering, as the hydrogel itself does not exert negative effects on the heart, while the bioresponsive injectable hydrogels provide optimal “on-demand” releasing media (Ifkovits et al., 2010; Eckhouse et al., 2014; Purcell et al., 2014; Vashist et al., 2017). Since most hydrogels contain ammonium persulfate (APS) and tetramethylethylene diamine (TEMED) (Ifkovits et al., 2010; Mironi-Harpaz et al., 2012; Yang H. et al., 2013; Eckhouse et al., 2014), some risk could be associated with using hydrogels as media for drug delivery. However, no significant cytotoxicity has been reported for the hydrogels in a short time frame (Ifkovits et al., 2010), and strategies have been developed to wash out APS and TEMED to minimize their potential toxic effects (Yang H. et al., 2013). HA hydrogels do not exacerbate heart function in MI animal models either (Ifkovits et al., 2010; Eckhouse et al., 2014).

### Intracoronary Delivery of TIMP3

In a model of cardiac ischemia/reperfusion (I/R) injury in pigs, intracoronary infusion of full-length human TIMP3 protein reduced infarct size and LV end-diastolic volume, and increased ejection fraction at 28 days post-I/R (Barlow et al., 2017; Thorn et al., 2019). Clearly, intracoronary delivery will require a larger amount of rTIMP3 compared to local myocardium injection in order to acquire the cardiac protective effect, while intracoronary infusion could result in off-target effects of TIMP3. However, it is easier to be translated from bench to bedside, because percutaneous coronary intervention (PCI) is an efficient and frequently used clinical therapeutic intervention for MI patients (Bates et al., 2016; Mehta et al., 2019).

### Application of TIMP3 in Restenosis and Vein Graft Neointima Hyperplasia

Although PCI is a safe and minimally invasive way for treating acute coronary syndrome, there are risks of intima hyperplasia and restenosis after the procedure, and TIMP3 has been employed to prevent and reduce the problems (Jukema et al., 2011; Hall and Agrawal, 2018). Implantation of the stent coated with recombinant adenovirus overexpressing TIMP3 (RadTIMP3, 20 μL of 10^9^ pfu/mL) in pig coronary arteries demonstrated localized overexpression of TIMP3 around the stent and increased apoptosis in the neointima and media (Johnson et al., 2005). RadTIMP3 decreased neointima area and expanded luminal area at 28 days after implantation. Late graft failure because of neointimal thickening and atherosclerosis is a serious problem in autologous saphenous vein coronary artery bypass graft surgery (CABG) (Wadey et al., 2018). Saphenous veins infected with RadTIMP3 (2.5 × 10^9^ pfu/mL) showed enhanced TIMP3 expression in the intimal and upper medial layers, and suppressed neointimal formation in human saphenous veins in organ culture and in pig interposition grafts *in vivo* (George et al., 2000). RadTIMP3-infected grafts had decreased intimal thickness at 28 days, and 3 months after the infection and implantation in autologous porcine arteriovenous interposition grafting (George et al., 2000, 2011). This function of TIMP3 in reducing the neointima formation and restenosis could be through inhibition of metalloproteinases, and induction of VSMC apoptosis through activation of FAS (George et al., 2000; Johnson et al., 2005; English et al., 2018). Therefore, TIMP3 holds the potential as therapy for restenosis and vein graft intimal thickening. Interestingly, a novel adenoviral vector with augmented vascular transduction and enhanced resistance to human serum neutralization has been developed (White et al., 2013), which could facilitate the translational and therapeutic application of TIMP3 in vascular diseases.

### Modification of TIMP3 Molecule to Optimize Its Therapeutic Properties

Besides the recombinant wild-type TIMP3 protein, utility of engineered TIMP3 molecule has also been explored. These modifications include addition of extra glycosylation sites, PEGylation (polyethylene glycol conjugation), and fusion with Fc, human serum albumin (HSA), antibody (Ab), or latency-associated peptide (LAP) (Chintalgattu et al., 2018; Alberts et al., 2019). To sequester TIMP3 in the extracellular space, TIMP3 could also be mutated (K26A/K45A and K42A/K110A) to disrupt its interaction with LRP-1 and the subsequent endocytosis (Doherty et al., 2016). Two engineered mutant TIMP3 molecules with extra glycosylation sites, TIMP3v2 (K22S/F34N) and TIMP3v82 (H55N/Q57T/K71N/E73T/D87N/K89T/R115T), have a higher expression yield in a Chinese hamster ovary cell line and longer rat serum half-life than the wild-type TIMP3 (Chintalgattu et al., 2018). TIMP3v2 or TIMP3v82 were injected into the infarcted myocardium after 3 h of LAD ligation in rats. Both could improve cardiac function at 3 days post-MI, but only TIMP3v82 had lasting effects until 7 days post-MI (Chintalgattu et al., 2018).

Tissue inhibitor of metalloproteinase 3 fused with LAP (LAP-TIMP3) has been shown to have a higher expression yield in 293T cells than wild-type TIMP3 (Alberts et al., 2019). The LAP-TIMP3 was designed with an MMP cleavage site (PLGL) in between LAP and TIMP3; therefore, the engineered TIMP3 was latent until it was released from LAP-TIMP3 by MMP1 (Alberts et al., 2019). The released TIMP3, containing a residual leucine at its N-terminal, could inhibit ADAM17 and ADAMTS4 but not MMPs, although in synovial fluid from patients with osteoarthritis, there was sufficient MMP activity to cleave LAP-TIMP3 and release active TIMP3 (Alberts et al., 2019), suggesting that LAP-TIMP3 may be useful for anti-inflammatory therapy.

Other peptides or synthetic peptides derived from TIMP3 could also be potential tools for treatment of diseases. A cyclic peptide derived from TIMP3 (61EASESLAG70 enclosed by a Cys at each end) targeting ADAMTS4 has been explored as a treatment for osteoarthritis (Zhang W. et al., 2018), further investigation is required for its potential usage in patients. Synthetic peptides corresponding to the loop 6 (aa 137−166) and tail region (aa 167−188) of TIMP3 are efficient in inhibiting angiogenesis which may be employed to treat diseases with uncontrolled neovascularization, such as choroidal neovascularization (Qi et al., 2013). Therefore, TIMP3 can be engineered to enhance the expression yield and to prolong its effects, as well as to inhibit specific metalloproteinases.

In summary, application of TIMP3 has been shown to be beneficial and effective in inhibiting adverse remodeling and improving heart function in MI and I/R animal models ranging from mice to pigs (Table 2). Overexpression of TIMP3 can also prevent and reduce restenosis and neointima hyperplasia after PCI or CABG. However, increased levels of TIMP3 resulted in considerable change in the cellular secretome through inhibition of ADAM10 and ligand-binding of LRP-1 (Scilabra et al., 2018). These suggest that the level of TIMP3 supplemented and the side effects related to the secretome change need to be monitored when TIMP3 overexpression or supplementation is applied. Local supplementation of TIMP3 has little to no side effects in non-target organs, however, most of these studies have been short-term, and the long-term effects need to be investigated, particularly those involving AAV-mediated overexpression. Intramyocardial injections can be done during the CABG procedure, by closed-chest myocardial injections guided by high-resolution echocardiography (Springer et al., 2005; Mattila et al., 2016), or catheter-based intracoronary delivery.

The reports on the application of TIMP3 in vascular diseases are limited to those pertaining to restenosis after PCI or CABG, as discussed above. TIMP3 can counteract inflammation and atherosclerosis in mouse models (Casagrande et al., 2012; Son et al., 2017); therefore, TIMP3 holds the potential as treatment for patients with atherosclerosis, although this requires further translational and clinical studies. In addition, given the protective role of TIMP3 in aortic aneurysm, and its dose-dependent function in angiogenesis, many potential therapeutic functions of TIMP3 remain to be explored. For instance, while pro-angiogenic function of TIMP3 would be beneficial in post-MI scenarios, its anti-angiogenic property at higher doses would limit the uncontrolled neovascularization in tumor growth and likely limit metastasis.

## Conclusion

In addition to the well-known MMP-inhibitory function of TIMP3, a variety of partners have been identified for TIMP3 revealing the diverse roles of this protein in various cellular and molecular functions, although the full spectrum of TIMP3 partners and the resulting outcomes requires further investigation. TIMP3 levels are mostly decreased in patients with cardiovascular conditions and deficiency of TIMP3 causes or exacerbates cardiovascular diseases partly mediated by elevated proteolytic activities (MMPs, ADAMs, and ADAMTSs). Supplementing TIMP3 in the myocardium can improve cardiovascular structure and function, although the mode, dose and site of administration will require rigorous monitoring and verification since excess TIMP3 can lead to undesirable outcomes.

## Author Contributions

Both authors listed have made a substantial, direct and intellectual contribution to the work, and approved it for publication.

## Conflict of Interest

The authors declare that the research was conducted in the absence of any commercial or financial relationships that could be construed as a potential conflict of interest. The reviewer AT declared a past collaboration with one of the authors ZK to the handling editor.
